# Regeneration of Zebrafish CNS: Adult Neurogenesis

**DOI:** 10.1155/2016/5815439

**Published:** 2016-06-13

**Authors:** Sukla Ghosh, Subhra Prakash Hui

**Affiliations:** Department of Biophysics, Molecular Biology and Bioinformatics, University of Calcutta, 92 A. P. C. Road, Kolkata 700009, India

## Abstract

Regeneration in the animal kingdom is one of the most fascinating problems that have allowed scientists to address many issues of fundamental importance in basic biology. However, we came to know that the regenerative capability may vary across different species. Among vertebrates, fish and amphibians are capable of regenerating a variety of complex organs through epimorphosis. Zebrafish is an excellent animal model, which can repair several organs like damaged retina, severed spinal cord, injured brain and heart, and amputated fins. The focus of the present paper is on spinal cord regeneration in adult zebrafish. We intend to discuss our current understanding of the cellular and molecular mechanism(s) that allows formation of proliferating progenitors and controls neurogenesis, which involve changes in epigenetic and transcription programs. Unlike mammals, zebrafish retains radial glia, a nonneuronal cell type in their adult central nervous system. Injury induced proliferation involves radial glia which proliferate, transcribe embryonic genes, and can give rise to new neurons. Recent technological development of exquisite molecular tools in zebrafish, such as cell ablation, lineage analysis, and novel and substantial microarray, together with advancement in stem cell biology, allowed us to investigate how progenitor cells contribute to the generation of appropriate structures and various underlying mechanisms like reprogramming.

## 1. Introduction

Traumatic injury to the central nervous system (CNS) in adult mammals would lead to significant pathology associated with long-term disability. Human statistics are frightening. The number of people living with spinal cord injury (SCI) has been estimated to be about 276,000 in USA alone. Each year, 1.4 million people sustain traumatic brain injury (TBI) resulting in an annual mortality of 50,000 people. The global scenario is far worse; approximately 500,000 people suffer SCI annually ([1]; the National SCI Statistical Center 2015, https://www.nscisc.uab.edu/; Christopher and Dana Reeves Foundation 2015, https://www.christopherreeve.org/).

Injury to the spinal cord triggers huge pathophysiological responses, followed by massive neuronal and glial cell loss. Since the adult mammalian CNS lacks any regenerative ability, the outcome of the tissue loss due to an injury causes long-term functional deficiency. No effective therapy is available to overcome these limitations in mammalian CNS. Any repairing strategy in mammalian CNS would require replenishment of lost cells, both glia and particularly neurons. Two therapeutic strategies to replace the lost neurons have been proposed: (a) transplantation of neural stem/progenitor cells and (b) inducing endogenous neural stem cell or progenitors. However, clinical implementation of either of these two strategies is not deliverable yet, because of the lack of understanding of the self-repair mechanism of the CNS. There are also several limitations of cell based therapies, such as determining the optimal cell type, nature, and time of cellular intervention and the assessment of appropriate functional recovery [2].

The general lack of regenerative ability is a characteristic of higher vertebrates like mammals, whereas regeneration is a very common feature among the lower vertebrates like fish and amphibians. Although the amphibians, like newt, salamander, and* Xenopus*, all have a high regenerative potential, the difficulty in breeding newt in captivity and the limited availability of genetic tools (only a few of them have recently been developed) proved these models relatively less appealing to the scientists. On the other hand, the zebrafish proves to be an excellent and popular model for a variety of reasons, for example, (a) very cost-effective maintenance and easy breeding with production of a large number of eggs/larvae and (b) easy amenability to various genetic analyses such as generation of transgenic lines, knockdown strategy like morpholino antisense technology, generation of mutants, and novel knockouts [3–6]. Another important property of this system is the transparency of its embryo, which allows us to undertake* in vivo* lineage tracking, and it could also be used as a behavioral and electrophysiological tool for the analysis of functional neural integration [7, 8]. Adult zebrafish has the amazing capacity of regenerating its spinal cord. It can repair its severed axons, replenish lost cells, induce neurogenesis after injury, and hence regain functional loss [9–13]. Understanding such a remarkable ability of endogenous regeneration in zebrafish, coupled with the new genetic tools and the commonality of its CNS architecture with that of other higher vertebrates, would be of major biomedical significance in inducing the regenerative potential in mammals including human. Thus, zebrafish could serve as an invaluable model to target functional regeneration of spinal cord in humans and to compliment SCI research based on other mammalian models. The array of reparative regeneration in this model also highlights the fact that the underlying cellular and molecular processes of regeneration have similarity with the developmental programs. In other words, at least some of the cellular processes of regeneration are also shared with the normal development of the particular organ [14, 15]. Thus, it is imperative to study the development of CNS and identify the important cellular and molecular cues to understand how it is constructed in the first place and then reconstructed.

## 2. Anatomy and Development of Zebrafish Spinal Cord

### 2.1. Neural Induction in Early Embryonic Development

Development of central nervous system begins with the formation of neural plate, an ectodermal derivative on the dorsal side of the embryo. Initial specifications of neural ectoderm or neural induction involve interaction between extrinsic signaling factors such as Bmp, Wnt, and Fgf and intrinsic signal such as transcription factors, the most important being soxB1 family members [16–18]. There is also an interplay between several secretory proteins such as chordin, noggin, and follistatin emanating from the organizer (equivalent is the shield organizer in zebrafish). These factors antagonize Bmp signaling which directs ectodermal cells to adopt a default neural fate. In zebrafish, several mutations have been generated like Dino (chordin), Cyclops (nodal related 2), Bozozok (Dharma), Swirl (Bmp-2), Snailhouse (Bmp-7), and so forth, and phenotypes of these mutants are corroborating the neural development through default pathway [19]. The neural ectoderm is also specified by soxB1 family members which are sox1 (a and b), sox2, sox3, and sox19 (a and b). Among these members,* sox2* and* sox19b* are both expressed in the presumptive CNS of the developing embryo and in neural progenitors of adult fish CNS [20–23]. Briefly, Bmp, Fgf, and soxB1 interactions are important in maintaining the neural stem cell pool in early embryonic development of zebrafish [24].

### 2.2. Formation of Neural Tube and Generation of A-P and D-V Pattern in Spinal Cord

Neural ectoderm once specified would form neural plate and then neural rod and eventually neural tube. Subsequent to neural plate formation, these plates would converge to form neural keel, followed by the formation of a solid structure referred to as neural rod, which eventually would become a hollow neural tube via secondary neurulation, finally forming brain anteriorly and the spinal cord posteriorly. The lining of the lumen of the neural tube is composed of pseudostratified neuroepithelial (NEP) cells. In early stage, the neural progenitor cells divide symmetrically to expand their pool of neural precursors and later increasing numbers of cells switch to asymmetric neurogenic division at the onset of neurogenesis in the neural tube. The NEP cells that undergo asymmetric division contribute to neurogenesis and transform themselves into radial glial cell, which is considered to be a neural stem cell population that exists throughout life in zebrafish unlike mammals [25, 26]. Furthermore, the property of asymmetric division is related to the fate of the daughter cells, the neurons being derived from the more apical daughters in asymmetric division, whereas the more basal daughters replenish the progenitor pool in zebrafish CNS [27, 28]. Generation of apicobasal polarity of neuroepithelial cells is crucial for CNS development. The proliferating neuronal progenitors shift their position from apical to pial side following a process called “interkinetic nuclear migration” (INM). The initiation of neurogenesis in zebrafish neural plate relies on the expression of several proneural genes, such as neurogenin 1 (*ngn1*) and achaete-scute 1 (*ascl1*), in the restricted cell population from which the nuclei of the primary neuronal network develop [29].

Studies have suggested that neural induction represents additional level of complexity, as FGF signaling promotes posterior structure by inhibiting Bmp in zebrafish [30]. Similar to other vertebrate models, specification of posterior CNS or spinal cord involves Fgf, Wnt, nodal, and retinoic acid (RA) signaling [17, 19].

**Figure 1:**
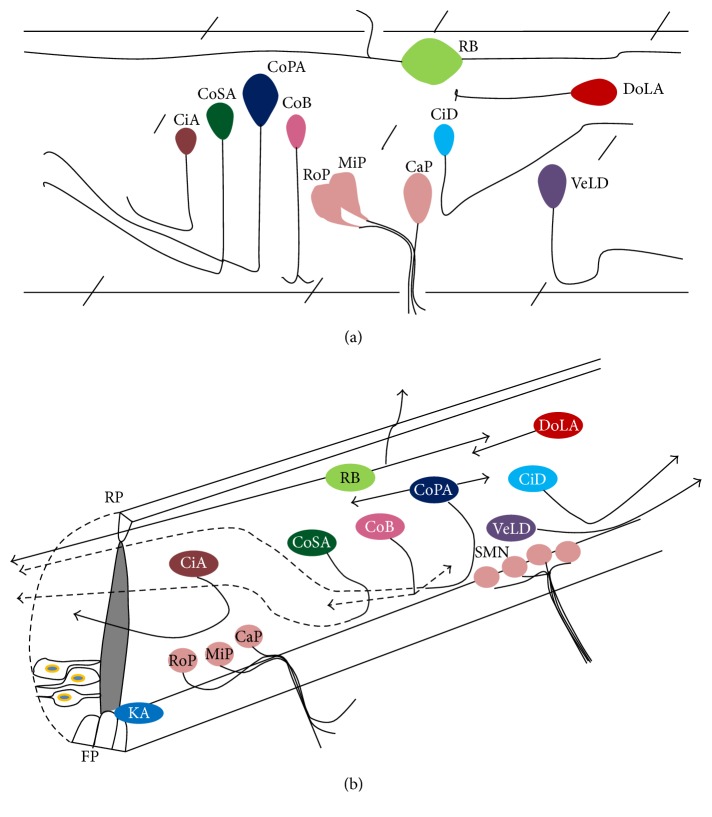
Schematic diagrams showing the anatomical location of different primary and secondary neurons in early embryonic and larval zebrafish spinal cord. (a) The drawing shows lateral view of spinal cord, anterior to the left. Showing locations of several types of primary and secondary neurons like Rohon-Beard (RB) sensory neuron, ventral longitudinal descending (VeLD) and commissural primary ascending (CoPA) interneuron and caudal primary (CaP), middle primary (MiP), and rostral primary (RoP) motor neurons are all primary neurons; commissural secondary ascending (CoSA) neurons are secondary interneurons. Dorsal longitudinal ascending (DoLA), circumferential descending (CiD), circumferential ascending (CiA), and commissural bifurcating (CoB) neurons are all interneurons. (b) represents spinal cord with interneurons and motor neurons of 1 day postfertilization embryo. Solid and hatched lines represent ipsilateral and contralateral axon projections, respectively. RB: Rohon-Beard sensory neuron; SMN: secondary motor neuron; KA: Kolmer-Agduhr neuron; VeLD: ventral longitudinal descending interneuron; DoLA: dorsal longitudinal ascending interneuron; CoPA: commissural primary ascending interneuron; CoSA: commissural secondary ascending interneuron; CiD: circumferential descending interneuron; CiA: circumferential ascending interneuron; CoB: commissural bifurcating interneuron; CaP: caudal primary motor neuron; MiP: middle primary motor neuron; RoP: rostral primary; RP: roof plate; FP: floor plate. Adapted and redrawn from [19].

Anterior-posterior (A-P) patterning of the neural tube would augment spinal cord formation, and within the cord there are additional strata of A-P patterning that is manifested by fin innervating motor neurons and positioning of motor neuron soma that innervate myotomes [31, 32]. Patterning of neural plate along the dorsoventral (D-V) axis resulted in specific location of floor plate (FP) cells, motor neurons towards the ventral side, and neural crest towards the dorsal side. Thus, FP cell is positioned in the ventral midline of the embryonic cord and comprises three longitudinal columns of cells and a single medial floor plate, flanked by two lateral floor plates on both sides [33–35]. The fate of medial floor plate is unknown, whereas lateral floor plate cells generate Kolmer-Agduhr (KA) neurons, a class of GABAergic neurons that has contact with cerebrospinal fluid (CSF) and may act as a proprioceptive position sensor [33, 36]. A subpopulation of olig2 positive cells originating from a distinct ventral cord precursor, referred to as P_MN_ cell, would usually give rise to motor neuron and oligodendrocyte and can also be maintained as radial glia that have stem cell-like character [37, 38]. NEP cells are proliferative in nature and can give rise to both neurons and glia. NEP cell is separated dorsally by nonneuronal roof plate (RP) cells whereas it is separated ventrally by FP cells. The sensory neurons are located in most dorsal positions; motor neurons occupy the ventral position and interneurons are at the intermediate position (Figure 1).

### 2.3. Neuronal Diversity and Formation of Primary and Secondary Neurons

Zebrafish spinal cord has both primary and secondary neurons. The primary neurons are large, born early, and fewer in number and undergo axonogenesis during the first day of development, whereas secondary neurons are smaller, born late, and higher in number (Figures 1(a)-1(b)). Secondary neurons have finer axons than their primary counterpart. Primary neurons include all different types of neurons such as sensory, motor, and interneurons. A large proportion of dorsally located Rohon-Beard (RB) sensory neurons die during development; only motor neurons and interneurons survive and persist in adult life. Secondary neurons consist of only interneurons and motor neurons. In the adult spinal cord, distinct types of interneurons are located along the D-V axis, although not all spinal interneurons develop at the same time. Commissural primary ascending (CoPA) and commissural secondary ascending (CoSA) interneurons are primary and secondary, respectively. CoSA interneurons have smaller soma with numerous thinner axons which extend their axon later but for a longer period of time than CoPAs.

Some dorsal longitudinal ascending (DoLA) and ventral longitudinal descending (VeLD) interneurons have extended growth cones by 18–20 hr of development whereas circumferential descending (CiD), circumferential ascending (CiA), commissural bifurcating (CoB), and KA interneurons do not extend growth cone until few hours later. Hence, CoPAs, DoLAs, and VeLDs are referred to as primary interneurons and CiDs, CiAs, CoSAs, CoBs, and KAs as secondary interneurons (Figure 1; [19, 39]).

Both primary and secondary motor neurons have overlapping yet distinct functions in adults [40]. The anatomical position of different neurons in the cord has been depicted in Figure 1. Both the FP and the RP cells synthesize and secrete several proteins and transcription factors that influence the fate and axonal trajectories. Relevance of cellular and molecular interaction during development and regeneration of spinal cord has been discussed in the latter section. Furthermore, the anatomy and the architecture of adult zebrafish CNS have been elegantly described and compared with mammalian CNS [41], so that its relevance to several human CNS disorders can be understood as described in Table 1.

## 3. Lesioning Paradigm: Advantages and Disadvantages

**Figure 2:**
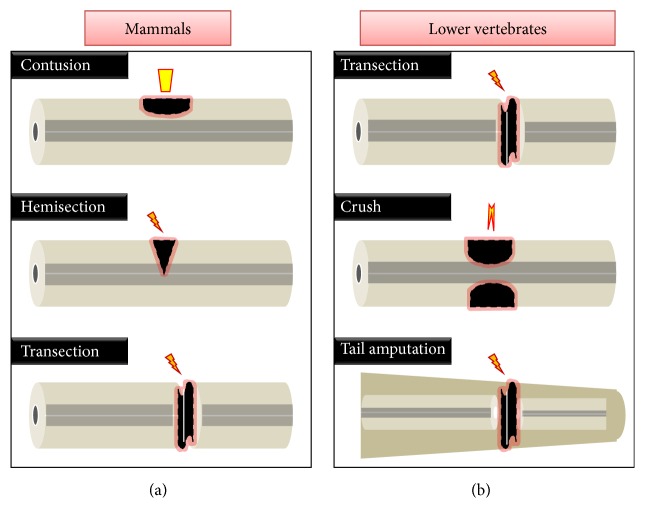
Spinal cord injury models used for the study of spinal cord regeneration in mammals and in lower vertebrates. (a) represents contusion injury, which is actually a compression injury inflicted by using a weight drop device; dorsal hemisection, that is, partial severing of the cord, usually ablates corticospinal tracts and part of the grey matter; transection injury with completely severed cord. (b) represents different experimental procedures such as transection (fully severed cord), crush (mechanical injury of cord with Dumont forceps), and tail amputation (removing caudal part of tail) for inflicting injury in lower vertebrates.

In the last two decades, the significant thrust of SCI research was on mammalian model, such as mouse, rat, or primates. The different injury protocols adapted in these regeneration incompetent models were directed towards understanding (a) the manner in which cells are affected or lost (white matter versus grey matter tissue loss), (b) what would the physiological consequence(s) be, and (c) how to relate the functional loss and outcome of a particular injury. A variety of lesioning protocols have been introduced in the beginning; later on, more and more refinement of modality has led to the evolution of stringent standardized protocols. The most widely used experimental methods are dorsal hemisection, contusion, and crush injuries (Figure 2(a)).

In case of lower vertebrates, the most widely used experimental protocol to study spinal cord regeneration is tail amputations (Figure 2(b)). Tail or caudal amputation involves complete removal of the caudal portion of the tail, where muscle, skin, bone, and cartilage are also removed along with the spinal cord. Many of the classical developmental biologists used this type of amputation model to study the regeneration of tail in the adults and larvae of newt, salamander, and* Xenopus* [42–46]. However, significant progress has been made in the understanding of the cellular basis of spinal cord regeneration in amphibians, based on tail regeneration, as there is a complete regeneration of tail along with the spinal cord and functional recovery. A major criticism about this model is that the regeneration of tail cannot be an appropriate model to study spinal cord regeneration in human, because of the variable nature of injury and absence of a tail structure. Furthermore, in order to extrapolate and use the information from lower vertebrates to humans, there is a call for developing a model comparable to that of a mammalian model. Other important injury paradigms are transection or resection that can be used in the context of mammalian regeneration. Transection refers to complete severing of cord which often develops spinal shock in humans, whereas resection refers to removal of a portion of tissue within the cord [47, 48]. Both of these modalities could be the ideal to study axonal regeneration since there is no axonal sparing, which some believe could augment regeneration in mammals [49]. Transection has also been used in urodeles to reveal the absence of glial scar, which is a major impediment to axonal regeneration in mammals [50], and to identify regeneration permissive environment during axonal regeneration [51, 52]. Moreover, transection model has been widely used in teleost to study axonal regeneration, revealing that most neurons with damaged axons would survive and contribute to regenerating axons [9, 10, 12, 53].

Amongst the entire collection of experimental paradigms mentioned here, compression and crush injuries are most widespread in mammals under experimental conditions and in human accidental injury conditions [54]. In search for an appropriate injury model to study the regeneration in teleost, we have successfully established standardized crush injury model in zebrafish, which is a comparable mammalian mode of injury [13]. Although it is technically difficult to introduce standardized crush injury compared to transection or tail amputation, as of now, it is the most suitable model to understand both the mammalian and the teleostean scenario. The outcome of crush versus transection injury differs. In the former condition, secondary degenerative response triggers axonal degeneration, whereas in the latter nerve tracts are severed almost immediately after injury. The most common experimental modality in mammals is contusion, which has been standardized and extensively refined for inducing variable lesions in mammals. However, standardizing and inflicting contusion injury in fish and amphibians got little relevance since the injury response to this particular injury is very minimal and hence would be difficult to calibrate and compare with mammals.

## 4. Regeneration of Spinal Cord following an Injury

After any insult to spinal cord in mammals, minimal functional recovery is observed. The primary damage is compounded by a complex series of cellular responses, such as loss of blood-brain barrier (BBB), causing inflammation due to invasion of blood cells in the injured site, cell death causing huge neuronal and glial loss, demyelination, and release of toxic myelin breakdown product, followed by axonal degeneration, generation of fluid filled cavity, and formation of fibroastrocytic scar. SCI in mammals results in a huge loss of astrocytes and oligodendrocytes (approximately 50%) and a much higher proportion of neurons [55]. Potential trigger for scar formation could be interleukin-1, Tgf-*β*, and fibrinogen [56]. Thus, combinations of causative factors are responsible for the development of neuropathology, lack of neural regeneration, and functional decline.

In contrast to mammals, injury responses in zebrafish spinal cord appear to be quite different resulting in repair of the injury and functional recovery [13]. Some of the cellular responses, which are different from the mammalian SCI, are as follows: (a) presence of a very brief inflammatory response which is controlled by different sets of genes [23], (b) presence of macrophages at the wound site which are probably involved in clearing myelin debris [11, 13, 53] and upregulation of anti-inflammatory M2 type macrophage related molecules, unlike mammals where accumulation of proinflammatory macrophages at the lesion site is observed which may be responsible for prolonged dieback of injured axons [57, 58], (c) very minimal cell loss due to necrosis and apoptosis after the injury (although apoptotic cell death is common to both mammalian and zebrafish SCI, the degree and extent of cell death are different in spatiotemporal pattern and involve upregulation of different sets of molecules, when compared with mammalian SCI), (d) proliferative response and extensive neurogenesis, and (e) generation of permissive environment for axonal regrowth. The major similarities and differences of cellular events and underlying molecular basis among mammals and zebrafish are also highlighted in our previous communications [13, 23].

## 5. Regenerative Responses: Injury Induced Proliferation

In mammals, cell proliferation in uninjured cord is very limited and provides low grade turnover of glial cells although a population of ependymal cells, which are expressing vimentin and parenchymal progenitor cells positive for olig2, are known to proliferate [59, 60]. In response to SCI, cell divisions occur between 1 and 3 days postinjury (DPI), and proliferation takes place at least in three locations such as the ependyma, the parenchyma, and the periphery [61]. In uninjured cord, divisions of ependymal cell are atypical but injury induces massive proliferation within 24 hr. Tracing of ependymal progeny revealed their migration to the injury site and primary contribution to becoming differentiated astrocytes and hence scar formation [59, 60, 62, 63], whereas an increased number of neurospheres* in vitro* from the injured cord suggest that neural progenitors proliferate in response to injury [64].

**Figure 3:**
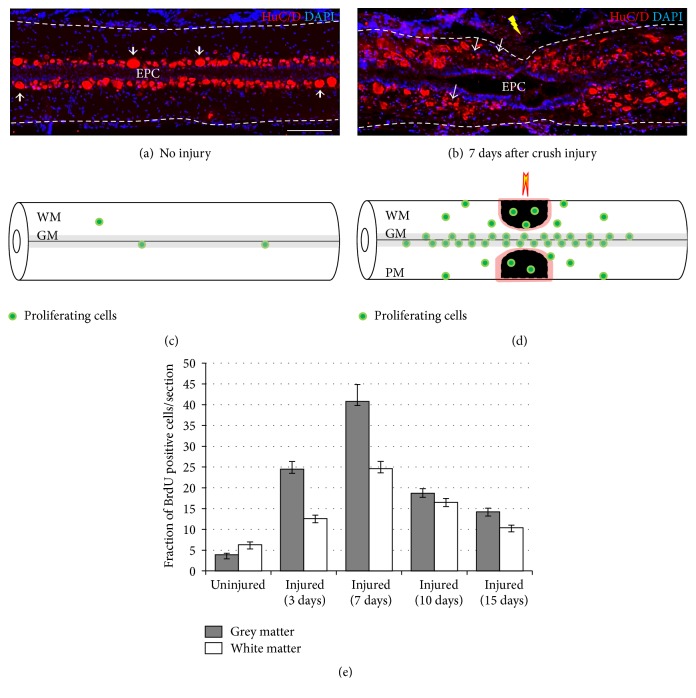
Analysis of BrdU incorporation and expression of neuronal marker HuC/D in adult zebrafish spinal cord. Longitudinal sections of uninjured (a) and a 7-day crush injured cord (b) immunostained with neuronal marker HuC/D. Neurons (thick arrow) are in the subependyma of uninjured cord and newly generated neurons (thin arrow) in the injury epicenter (yellow arrow) of 7-day injured cord. EPC marks the ependymal canal of the cord. (c) Schematic diagram showing locations of proliferating cells in adult uninjured spinal cord (c) and injured cord (d) after BrdU incorporation study. WM: white matter; GM: grey matter. (e) Quantification of proliferating cells after crush injury in zebrafish spinal cord (adapted from [13]). Scale bar = 200 *μ*m (a, b).

Uninjured zebrafish spinal cord however appears to be relatively quiescent and slowly dividing, where BrdU incorporation is documented predominantly in the ventricular zone of the spinal cord [12, 13]. Upon injury, the proliferation increases dramatically both in crush and in transection. In the crush injured spinal cord, the proliferation begins around 3 DPI in the region which is rostrally and caudally a short distance away from the injury epicenter, followed by an elevated number of proliferating cells at the injury epicenter in 7 DPI. BrdU incorporation occurs both in white matter (WM) and in grey matter (GM), suggesting proliferation in both of these compartments (Figures 3(d) and 3(e)). The total number of BrdU positive cells in GM is far higher than that of the WM at the time point of peak proliferation (7 DPI) and the predominant proliferation zone remains at the ventricular zone. Time course analysis of injury induced proliferation after crush injury is represented in Figure 3(e). The proliferation is a very controlled event which gradually decreases in time and proliferation rate comes back towards the normal level seen in uninjured cord. However, the temporal sequence of injury induced proliferation may vary between crush and transection injury. BrdU incorporation studies can further identify two different populations of proliferating cells, like slow-dividing intensely labeled cells which are actually representing the quiescent population and others that are fast-dividing and hence loosely labeled cells which are probably transit amplifying cells and are GFAP negative [13]. A vast majority of the BrdU positive cells in the regenerating cord are radial glia around the ependyma, newly born neurons, and macrophages as identified by expression of cell specific markers, BrdU labeling, and ultrastructural analysis [13, 65]. HuC/D positive neurons are around the ependyma in uninjured cord (Figure 3(a)) and small newly born neurons are present near injury epicenter of the injured cord (Figure 3(b)).

### 5.1. Proliferation and Cell Cycle Regulation

**Figure 4:**
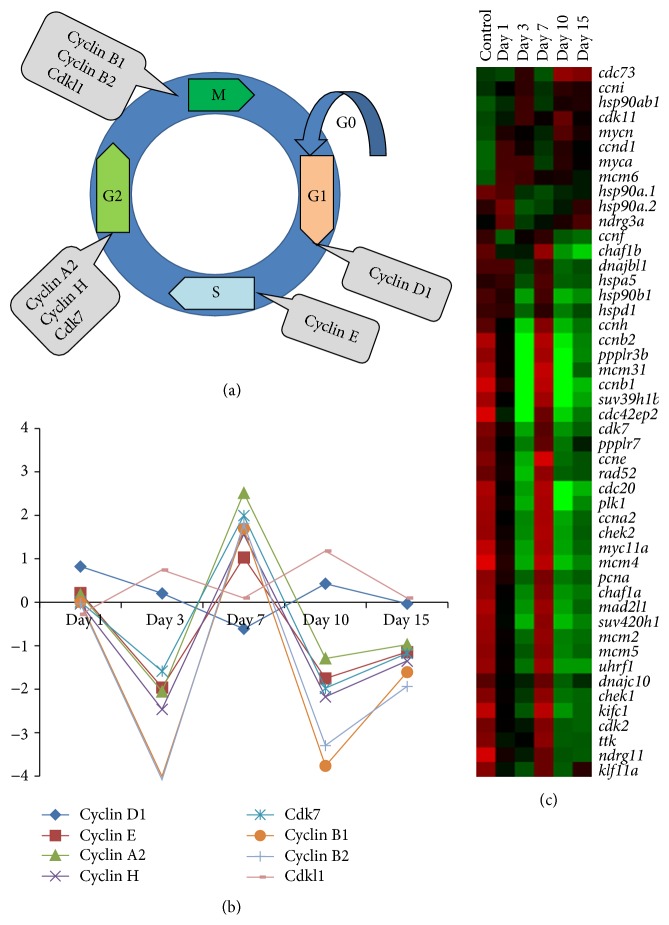
Analysis of cyclins and Cdks during spinal cord regeneration. (a) Schematic diagram shows cell cycle regulatory genes expressed during adult zebrafish spinal cord regeneration [23]. (b) Expression pattern of various cell cycle regulatory genes based on microarray analysis during spinal cord regeneration [23]. Several cyclins associated with different cell cycle phases are shown such as cyclin D1 (G1), cyclin E (S), cyclin A2 (G2), cyclin H (G2), Cdk7 (G2), cyclin B1 (G2-M), cyclin B2 (G2-M), and Cdkl1 (G2-M). (c) Heat maps of cell cycle regulatory genes expressed during regeneration showing different temporal pattern.

In zebrafish SCI, it is important to decipher how cell proliferation would contribute to neurogenesis and successful regeneration of spinal cord. We shed some light on how cell proliferation, cell cycle entry/exit, and neurogenesis are highly coordinated to restore the structure and function of injured spinal cord. Since injury induced proliferation is associated with cell cycle regulation, we observed as many as 48 differentially regulated genes, directly associated with cell cycle (Group A); conceivably, many are cyclins and cdc/cdks (Figures 4(b) and 4(c), [23]). Another group of 30 genes (Group B in [23]) indirectly control cell proliferation, which include both positive and negative regulators of cell cycle either upregulated or downregulated. Expression of only a handful of these genes in Group A and Group B is validated such as* ccnd1*,* ccnb1*,* ccne*,* cdk2*,* tgfb1*, and* neurod* [23]. Almost all these genes in Group A and only a few in Group B are expressed in uninjured cord but are upregulated in 7 DPI cord, when rate of proliferation is the highest compared to uninjured cord. Interestingly, these genes are not expressed in any other injury time points. Our previous observation based on cell counts of colocalized BrdU/H3P cells showed that, in uninjured cord, only 1 and 2% of cells are in M-phase and S-phase, respectively, compared to high percentage (97%) of cells in G_0_-G1-phase. In injury, the percentage of cells both in S-phase (5%) and in M-phase (12%) increases greatly in 7 DPI cord [23]. cDNA array analysis data reflects the cell cycle control before and after injury; schematized expression of different genes in different cell cycle phases during regeneration is shown in Figure 4(a). Therefore, genes involved in G1-S-phase transition are selectively upregulated either in 3 DPI (*ccnd1*,* ccni*, and* myc*, early proliferative stage) or in 7 DPI (*cdk2*,* cdk7*,* ccne*, and* ccnh*) cord, whereas all 3 genes associated with S-phase are upregulated in 7 DPI cord (*ccna2*,* pcna*, and* uhrf1*), when the highest number of proliferating cells is in S-phase as confirmed by BrdU incorporation study (Figure 4(a); [13]).

Cdk4 and cyclin D1 are involved in progenitor cell expansion and are inhibitor(s) of neurogenesis in developing mammalian CNS [66]. Changes in cell cycle length are associated with determination of cell fate and neurogenesis and longer G1-phase is characteristic of neurogenic progenitor [67]. In primate cortex, higher proportions of neurogenic divisions are regarded as progenitors with longer G1-phase [68]. We observed in injured zebrafish cord that* ccnd1* is expressed very early in 1 DPI cord, when progenitor expansion is probably required but neurogenesis does not take place at this early regenerative phase. In* Xenopus* spinal cord, ccdx, another type of cyclin D, is expressed in ventral P_MNs_ and is required for generation of differentiated motor neurons. These findings may underline the importance of specific cyclins and their roles in the maintenance of specific progenitor pools of neural cells in the CNS [69, 70]. Some of these molecules mentioned above may have conserved role in CNS development of different species, but specific evidence in regenerating CNS is necessary for better understanding of the mechanism of initiation and maintenance of proliferative response. Among the several cyclin D1 interactors, important ones are cdk4 and cdk6 along with Kip/Cip family of inhibitors [71]. However, cell cycle machinery controls not only proliferation but also cell cycle dependent movement like interkinetic nuclear migration (INM) in neural progenitors, at least in zebrafish retina [72]. Both neuroepithelial and radial glial cells demonstrate INM as they progress through the cell cycle, in which the nucleus translocates to the luminal surface where mitosis occurs [28, 73, 74].

In injured zebrafish cord, at least 10 out of 12 identified genes involved in G2-M transition (*ccnb1*,* ccnb2*,* cdc20*,* kif11*,* mcm6*,* mcm2*,* mad2l1*,* ttk*,* plk1*, and* kifc1*) are also upregulated in 7 DPI, when the number of proliferating cells is the highest. Since a greater percentage of cells are expressing M-phase marker in 7 DPI cord, it suggests that many of the proliferating cells are indeed going through mitosis (as confirmed by a mitosis marker H3P) and differentiate as neurons (as these cells are neuroD positive) in the regenerated cord [13]. Several cell cycle regulators are commonly expressed in different regenerating systems like fin, retina, heart, and spinal cord. These are* plk1* (polo kinase 1),* ttk*/*mps1* (monopolar spindle 1, a kinase required for mitotic check points regulation),* cdc20*,* ccna2*, and* kif11* [23]. Among these common genes, three genes, namely,* plk1*,* cdc20*, and* ccna2*, are M-phase regulators and all three showed upregulation with high fold change in 7 DPI cord. They may have role(s) in regulating cell proliferation. Differential temporal expression pattern of proliferation markers like PCNA (all phases), BrdU incorporation (S-phase), and H3P (M-phase) in regenerating cord highlights the complex yet very well coordinated cell proliferation required during the regeneration of cord. These proliferative events may involve a similar mechanism during development of neural tube. Recently, the role of another common gene,* kif11*, has been elucidated in developing zebrafish spinal cord, where it functions in spindle pole separation during mitosis, and hence radial glia are arrested or slowed in mitosis in* kif11* mutant fish [75]. Loss of* kif11* causes accumulation of radial glia in mitosis and mutant embryos display monastral spindles, a distinctive feature of mitotic arrest. Among the common genes, another important gene is* mps1*/*ttk*, the expression of which has been documented in different regenerating organs such as fin, regenerating heart, and retina along with regenerating spinal cord [23, 76–78] where all the progenitors like blastemal cells, cardiomyocytes, and neural progenitors are intensely proliferating. Moreover, involvement of these common molecules in controlling cell proliferation/cell cycle in these different tissues highlights the possibility of existence of a conserved mechanism in all regenerating systems mentioned. Expression of these common molecules in a selected time frame and in a particular tissue refers to their involvement in a particular event. More detailed analysis involving lineage tracer and functional assay of these cell cycle regulators could further improve our knowledge in understanding the role of cell cycle regulators in CNS development and regeneration.

## 6. Neurogenesis

### 6.1. Neurogenesis in Adult CNS

**Figure 5:**
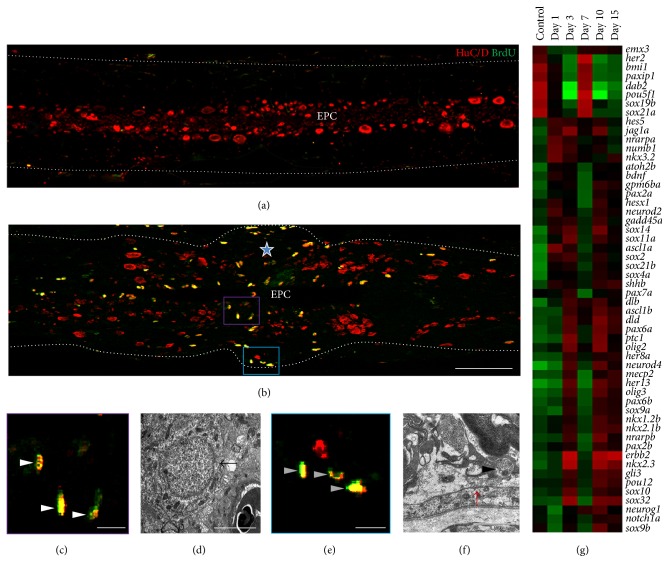
Proliferation and neurogenesis in zebrafish spinal cord. (a-b) Longitudinal sections of uninjured (a) and injured (b) cord showing Hu/BrdU colocalization around ependyma, indicating injury induced proliferation and neurogenesis. Star demarcates the injury epicenter; EPC: ependymal canal. (c) Violet boxed area in (b) represents Hu/BrdU colocalised cells (white arrowheads) in 7 DPI cord at higher magnification. (d) represents ultrastructure of a newly born neuron at the injury epicenter of an injured cord, with high nucleocytoplasmic ratio and very few organelles; the arrow points at the boundary of cytoplasm. (e) Blue boxed area in (b), showing subpial neuronal precursors stained with Hu/BrdU (grey arrowheads) in injured spinal cord at higher magnification. (f) An ultrastructural view of subpial neuron (black arrowhead) near the pial membrane (red arrow). (g) Heat maps representing genes related to neurogenesis differentially expressed during regeneration of zebrafish spinal cord. Scale bar = 200 *μ*m (a, b), 5 *μ*m (c, e), 2 *μ*m (d), and 1 *μ*m (f).

Adult neurogenesis has been demonstrated in all vertebrate species although there is a decreasing trend both in the number of proliferating zones and in the number of newborn cells in more evolved species such as mammals. A more precise and comprehensive knowledge of adult neurogenesis under both physiological and pathological conditions could be a breakthrough in developing new therapeutic strategies for neurodegenerative disorders, SCI, and stroke. Once again, analysis of adult neurogenesis before and after SCI in zebrafish is a necessary prerequisite to understand the cellular and molecular basis of the enormous plasticity of CNS in this species. Here, we discuss the characteristic of neurogenesis particularly in adult spinal cord. Zebrafish cord displays a huge surge of proliferation following injury and thus continues to produce neuronal precursors, which would migrate and differentiate into functional mature neurons. In adult fish brain, the presence of proliferating cells in the ventricular zones was published long ago [79, 80] and, subsequently, the presence of proliferating zones in the different regions of brain in most other teleost species has also been reported [81–86], although proliferative zones differ in their rate of generation of new cells [86]. Adult neurogenesis in zebrafish brain has been extensively studied, where constitutive neurogenesis occurs so that a net increase in the number of neurons with age can take place. Both adult mammalian [60, 87] and zebrafish spinal cords do not harbor a constitutively active neurogenic zone. We observe very few BrdU positive cells in uninjured cord (Figure 5(a)). Upon injury, restorative neurogenesis can be induced in different regions of zebrafish CNS, such as telencephalon and spinal cord [12, 13, 86]. In contrast, injury to neurogenic site in mammals resulted in emigration of progenitors and generation of newly born neurons, but they fail to survive [88], while others reported that proliferating progenitors only generate glial cells [60]. Interestingly, progenitor cell can produce neurons either* in vitro* or when grafted into neurogenic region of brain like adult dentate gyrus [89].

### 6.2. Neurogenesis in Zebrafish Spinal Cord

Fish brain is capable of neurogenesis as mentioned by many authors [21, 25, 90]; however, studies on neurogenesis in spinal cord are still few and far between. More experimental lines of evidence are necessary to determine the origin, fate, and differentiation of proliferating precursors and their survival and integration into the neural circuitry. Analysis on zebrafish spinal cord suggests that there is presence of neural progenitor/stem cells and the proliferation after injury indeed generates new neurons and at least a population of these newly born neurons survives and integrates into the regenerating cord. These inferences are primarily based on the evidence that a large proportion of proliferating cells do express several neuronal and progenitor cell markers like HuC/D, NeuroD, and Sox2 (Figure 5; [13, 91]). Proliferation occurs in both the injury epicenter and the adjacent areas, and it is widespread in ependyma and subependyma. In 10 DPI cord, many cells are BrdU and HuC/D positive, suggesting that these proliferating cells are newly born neurons as confirmed by histological, ultrastructural, and immunohistological analysis (Figures 5(b), 5(c), and 5(d)). However, circumstantial evidence points towards the fact that the ventricular region of ependyma generates neurons; subsequently, these newly born neurons settle in the adjacent subependymal zone as these cells are Hu/BrdU and Hu/NeuroD positive [13]. Different types of neurons that can regenerate in spinal cord are dorsal* pax2* expressing interneuron,* dbx* expressing interneuron V_0_/V_1_, V_2_ interneuron,* islet1/2* expressing motor neurons, and serotonergic interneurons, all generated after either crush or transection injury [12, 13, 23, 92–94].

Previous analysis of the anatomical profile of axonal growth and function suggested that neurons within reticular formation (RT), magnocellular octaval nucleus (MaON), and nucleus of the medial longitudinal fascicle (NMLF) grew their axons and represent the most regenerative neurons (Table 1; [53]) and are involved in the descending control of swimming behavior [95]. It has been demonstrated that the projection axons can influence cell proliferation in appropriate target areas. The signals from descending axons from brain to the spinal cord actually regulate spinal progenitor cell proliferation and differentiation during development. One great example of such a highly coordinated signal is the dopaminergic projection from brain to spinal cord. Axons from diencephalic dopaminergic neurons supply spinal cord during neuronal differentiation and serve as the only source of dopamine in zebrafish spinal cord in 2-day postfertilization embryo, a stage that coincides with generation of spinal motor neurons [96]. Recently, Reimer et al. [97] also showed that dopamine from brain promotes generation of motor neurons in developing zebrafish spinal cord at the expense of V_2_ interneuron. Thus, dopamine released from descending tracts is a powerful regulator of spinal neurogenesis. Endogenous dopamine promotes generation of spinal motor neurons by attenuating the response of progenitors to Shh signaling.

### 6.3. Subpial Neurogenesis

There is little evidence of adult neurogenesis outside hippocampal subgranular zone and forebrain subventricular zone in adult mammalian brain. However, in search for latent neurogenic potential, some evidence has come from nonproliferating region of brain like neocortex, where reparative neurogenesis can be observed after selective ablation of specific neuronal projection [98]. Ohira et al. [99] reported another source of cortical neuron in adult rat brain, where these progenitors are found in the subpial region of rat neocortex and are a small number of dividing cells that can be activated after ischemic injury. Transient ischemia induces proliferation of interneuron precursor in the subpial region, followed by migration in the lower layers of cortex. These precursors differentiate into GABAergic neurons and are expressing GAD67, a GABA synthetic enzyme [100]. In developing dentate gyrus, a temporary neurogenic region adjacent to meninges has also been identified [101]. A detailed analysis of cell proliferation in subpial layer and association of these neurogenic precursors with Bergmann glial end feet has been described in an elegant study in perinatal rabbit cerebellar cortex [102]. Similar to mammals, in the adult zebrafish forebrain, subpial locations of neurogenesis do exist in the early cerebellar external granular layer [103]. These cells do express neuronal markers like HuC/D, NeuroD, and Ngn and migration of these cells occurs in different waves towards different region of brain. We have observed subpial location of cells in the adult zebrafish spinal cord; there may be an increase in these proliferating neuronal precursors following SCI (Figures 5(b), 5(e), and 5(f); [13]). So, identifying the signals which trigger subpial neurogenesis and understanding the proliferation, migration, and differentiation of these newly generated neurons could augment potential therapeutic strategies to stimulate neurogenesis after stroke and SCI and in other neurodegenerative disorders.

## 7. Radial Glia as Neuronal Progenitor

We have observed that proliferation zone can give rise to various neuronal cell types, so it is important to uncover the identity of CNS stem/progenitor cells. In the rodent, bird, and reptiles, the progenitor/stem cell population exhibit distinct glial phenotypes of radial glia or astrocytes, most of which are in contact with the ventricular lumen, and this particular cell type has been identified as the source of new neurons [104–106]. Radial glia in mammals do not exist after birth, giving rise to ependymal cells and astrocytes which may retain the stem cell-like characters, whereas adult zebrafish brain and spinal cord harbor radial glia retaining embryonic characters. These cells do express genes similar to their embryonic counterparts like glial fibrillary acidic protein (GFAP), S100*β*, brain lipid-binding protein (BLBP), glial high affinity glutamate transporter (GLAST), and vimentin [12, 13, 65, 86, 91]. Radial glial cells have the properties of true stem cells, since these cells can self-renew and are capable of generation of different cell types like neuron and glia. Using Cre-loxP recombination based lineage tracing analysis in adult zebrafish, it has been demonstrated that ventricular radial glia function as neuronal progenitor after injury in the telencephalon [90]. However, clonal analysis of these glia from constitutively neurogenic region of telencephalon indeed exhibited properties of stem cells such as self-renewal and generation of different cells types like neuron and glia [107]. In the regenerating zebrafish cord, we observed that a majority of cells around the ependymal canal are sox2 positive radial glia [91]. Many of them are proliferating, and some are slow dividing, hence quiescent and capable of self-renewal, while others are transient amplifying cells and are considered to be neuronal precursors similar to developing and adult mammalian CNS [13, 65, 108]. Lineage tracing analysis using* olig2*:GFP transgenic zebrafish line reported the presence of slow proliferating radial glia that give rise to motor neurons after transection injury [12]. These regenerated motor neurons are generated from olig2 positive radial glia and exhibit markers like HB9 or islet1/2; upon terminal differentiation, these neurons express ChAT and SV2, suggesting that these motor neurons are probably integrated into the spinal circuitry. Furthermore, regenerated motor neuron in the spinal cord can be labeled retrogradely from the muscle, signifying that some of these grow axon out of the ventral root, a precondition for functional motor neuron. Morphological characteristic of radial glia appears to be similar throughout the CNS, while there is heterogeneity within the radial glial population as observed by using different glial markers like BLBP, GLAST, and GFAP [91]. There are distinct regional differences in their gene expression as these radial glial cells give rise to different types of neuronal progeny after injury. For example, in the ventral spinal cord, there are P_MN_-like radial glia in lateral position, with overlapping expression of* nkx6.1*,* pax-6*, and* olig2* that generate motor neurons in regenerating cord similar to P_MN_ domain of developing cord [92, 93]. The V_2_ interneurons, which are dorsal to P_MN_-like domain, express* vsx1*. Thus, progenitor domains of motor neuron and V_2_ interneuron are thought to be spatially similar in both regenerating and developing cord. Moreover, in postembryonic regenerating cord, medial radial glia express* dbx1* and contribute to neurogenesis [109].

Radial glia show a radial phenotype with long radial processes, the end feet of which touch the pial surface and soma contributing to the ependymal lining of ventricle. Thus, these cells retain the bipolar morphology of their neuroepithelial ancestor that can serve as neural stem cell in the vertebrate nervous system. Their proliferation is tightly regulated in order to produce appropriate number of neurons and glia in neural tube. Recently, a zebrafish mutant has been generated to show that a particular gene* kif11* is controlling generation of radial glia, where a high number of M-phase radial glia in the ventricular region have been demonstrated [75, 110]. During development, proper radial glial division is crucial in generation of oligodendrocyte, secondary interneurons, and motor neuron as evidenced in loss-of-function mutation of* kif11*, where reduction of these phenotypes is observed. Evidence is there to suggest that the expression patterns of several genes in regenerating spinal cord are similar to developing neural tube. Expression of* kif11* is one of the 29 common genes involved in different regenerating structures [23] and is upregulated in 7 DPI cord. This data provides indirect evidence of involvement of* kif11* in regenerating cord, where* kif11* may be controlling proliferation and maintenance of radial glial phenotype and may influence neural stem cell division as well as generation of neurons similar to the developing neural tube. However, in the future, more direct experimental evidence is required to reconfirm this hypothesis. All these experimental data prove that radial glia are the major source of regenerated neurons in the lesioned spinal cord and brain.

## 8. Transcription Factors Regulating Neurogenesis

Generation of neuronal diversity is a crucial step in the development and regeneration of zebrafish spinal cord. We have summarized the role of transcription factors in assigning progenitor domain in developing cord and also the involvement of known transcription factors in neurogenesis and neuronal differentiation in regenerating cord.

### 8.1. Neuronal Specification along D-V Axis in Developing Spinal Cord

**Figure 6:**
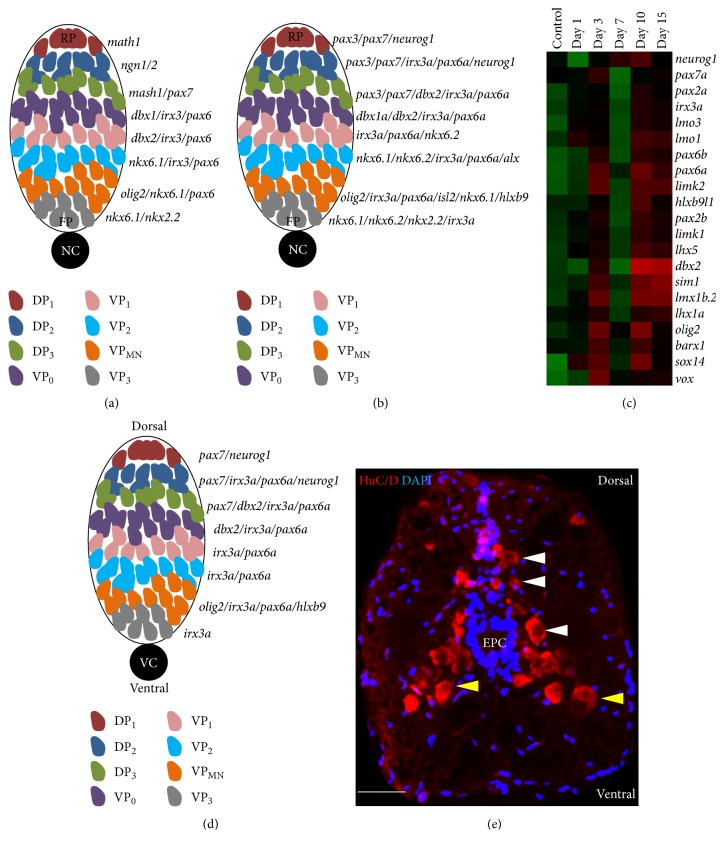
Expression and regulation of transcription factors involved in neuronal specification. (a) Dorsoventral patterning in the vertebrate neural tube [117]. (b) Dorsoventral patterning in the zebrafish neural tube [114]. (c) Heat map represents differential expression of transcription factors related to dorsoventral repatterning of cord in regenerating adult zebrafish spinal cord [23]. (d) Based on microarray data and expression profiling, predicted model of dorsoventral patterning in the adult regenerating zebrafish spinal cord. (e) A transverse section of 15 DPI adult spinal cord immunostained with HuC/D antibody showing regenerated neurons with characteristic dorsoventral localization (white arrowheads and yellow arrowheads indicating dorsal and ventral neurons, resp.). RP: roof plate; FP: floor plate; NC: notochord; DP: dorsal progenitor; VP: ventral progenitor; VC: vertebral column. Scale bar = 10 *μ*m (e).

The spinal cord development begins with a population of neural progenitors which initially assemble together into distinct domains along the dorsoventral (D-V) axis in response to several local signals and each of them would give rise to a different type of neuron. There are five distinct progenitor domains in the ventral spinal cord, namely, P_3_, P_MN_, P_2_, P_1_, and P_0_ (Figure 6). The most dorsal domain of ventral spinal cord is P_0_ domain expressing dbx1. The neurons generated from this domain are V_0_ interneurons, while dbx2 expression is seen in these cells as well as in V_1_ interneuron progenitors [94, 111]. The P_0_ progenitors produce both excitatory and inhibitory neurons. In zebrafish, these V_0_ neurons are all with commissural axons but generate both excitatory and inhibitory neurons. The transcription factors* evx2* and* pax2* mark the V_0_ excitatory and inhibitory neurons, respectively, and, unlike mouse, P_0_ neurons are not labeled by* pitx* in zebrafish. The P_0_ progenitors are heterogeneous and there is a temporal order of neuronal differentiation. The earliest phase generates only excitatory neurons, followed by the late phase when both V_0_ excitatory and inhibitory neurons are continuously produced.

The P_2_ progenitor domain resides in the ventral spinal cord and generates two interneuron subtypes, V_2a_ and V_2b_. The P_2_ progenitors with high level of* vsx1*/*chx10.1* expression represent an intermediate stage committed to become pair producing progenitors. Almost all V_2_ neurons are produced by pair generating progenitors that divide once to produce V_2a_/V_2b_ pairs [112].

The most ventral domain of the ventral cord produces V_3_ interneurons and motor neurons.* Olig2* transcription factor is expressed in P_MN_ and P_3_ [113].* Nkx2.2* is expressed in lateral floor plate, the V_3_ interneuron progenitors (P_3_) that arise ventral to P_MN_ progenitors, whereas* nkx6.2* expression includes the ventral half of spinal cord including floor plate [114].

*Islet1/2* and* hlxb9* are expressed within* nkx6.1* and* nkx6.2* domain, suggesting their role in differentiation of motor neuron. Both in teleost and in amniotes,* pax3* is the most dorsally expressed gene, whereas* pax6* is the most broadly expressed gene throughout the D-V axis with the exception of floor plate (FP) and roof plate (RP). In the ventral spinal cord,* pax6* expression includes V_0_-V_2_ interneuron and motor neuron progenitors, that is, P_0_-P_2_, P_MN_ [115]. The most recent depiction of transcription factors expressed in zebrafish ventral spinal cord is mentioned in Table 2, adapted from a study by England et al. [116].

Based on several literatures [114, 117, 118], we have schematized the expression of several transcription factors that are involved in neuronal specification along the D-V axis of spinal cord during the development in zebrafish and other vertebrates (Figures 6(a) and 6(b)). These results indicate that spinal progenitor gene expression patterns are largely conserved in zebrafish and amniotes.

### 8.2. Generation of Distinct Type of Motor Neuron and Interneuron

Distinct types of neurons are located along the D-V axis of the embryonic and larval spinal cord (Figure 1) and the expression of a few cell specific molecular markers allows us to identify these neurons as discussed below. Primary motor neurons express* islet1* and are required for assigning motor neuron fate in mouse, whereas at a later phase primary motor neuron downregulates expression of* islet1* and expresses another related member,* islet2* [119]. In zebrafish,* islet1* is required for both primary and secondary motor neuron formation and appears to mediate a switch between motor neuron and interneuron fates in P_MN_ domain. However, expression of* islet1* may inhibit interneuron formation. Zebrafish primary motor neuron coexpresses* islet1* and* lhx3*, whereas VeLD interneuron expresses* lhx3* but not* islet1* [118], although they are derived from the P_MN_ domain. Secondary motor neurons and VeLD show a segmental distribution pattern.* Islet2* is required for normal development of caudal primary (CaPs) motor neurons and when* islet2* function is knocked down these cells develop VeLD-like morphology and express GABA rather than Ach (a primary motor neuron neurotransmitter; [19]). Detailed analysis of* islet1* and* islet2* expression in zebrafish spinal cord demonstrated that although* islet2* is expressed only in CaPs, either* islet1* or* islet2* is adequate for CaP subtype identity. Similarly,* islet1* expression is maintained in middle primary (MiPs) but not in CaPs and* islet1* is not required for subtype specification of MiPs.* Islet1* is the first gene to be expressed in primary motor neurons along with pattern forming genes like* olig2* and* nkx6.1*. The P_MN_ domain also generates interneurons like VeLD, KA, and CiD. Other analyses indicate that DoLA expresses* spt* along with* islet1*,* islet2*, and* islet3*, CoSA expresses* pax2a* and* evx1*, and VeLD expresses* lim3* [118], whereas MiP and rostral primary (RoPs) motor neurons express* islet1* and CaP and VaP express* islet2*.

## 9. Transcription Factors Regulating Neurogenesis in Regenerating Spinal Cord

There are clear indications that zebrafish spinal cord displays dorsoventral transcription factor identities in the regenerating spinal cord, which resembles or recapitulates the expression pattern of several transcription factors involved in neuronal specification in the developing cord. Several examples are discussed here, like the specification of motor neuron (*olig2* ^+^,* pax6* ^+^, and* nkx6.1* ^+^) in ventral spinal cord, whereas V_2_ interneurons originate in P_2_-like domain and are* nkx6.1* ^*+*^ and* pax6* ^*+*^ but* olig2* ^−^ (dorsal to and contiguous with motor neuron progenitor domain). Using a Tg(*vsx*1:GFP) line, it has been demonstrated that Tg(*vsx1*:GFP)^+^ cells that emerge from P_2_-like progenitor domain are generated after injury.* Pax2* ^+^ neurons are also newly produced after the injury and they are distinct from Tg(*vsx1*:GFP)^+^ interneuron, although the origin of* pax2* interneuron is not known yet [93]. The transcription factors involved in D-V specification are differentially regulated in regenerating cord and represented in the heat map (Figure 6(c)).

To understand the molecular basis of neurogenesis in regenerating cord, we have analyzed cDNA microarray data, where at least 54 genes involved in neurogenesis and neuronal differentiation are differentially regulated (Figure 5(g); [23]), and among them 41 genes are transcription factors. We have validated the expression of several transcription factors that are upregulated during the regeneration and are particularly associated with neurogenesis. These are* pax6a*,* dbx2*, and* neuroD* [23]. We have discussed that the expression of many transcription factors is responsible for specification of neuronal subtype identity along the D-V axis of developing cord. Similarly,* dbx*,* irx3*, and* pax6* involved in V_0_ and V_1_ patterning are expressed in the regenerating zebrafish cord, whereas* olig2*,* pax6*,* hlxb9*, and* islet2* involved in P_MN_ domain are upregulated. Neuronal population in dorsal domain is specified by mash, math, neurogenin, and LIM homeobox genes in developing vertebrate cord, whereas we observe* ngn1*,* lim1*, and* lim3* upregulation in regenerating cord [23].

Several genes like* her2*,* dab2*,* pou5fl.1*,* emx3*,* bmi*,* paxip*,* sox19b*, and* sox21a* are upregulated in 7 DPI cord where proliferation is very high, and some of these transcription factors may be associated with neurogenesis, such as* pou5fl.1* [91]. Involvements of* sox19*,* her2*, and* dab2* have been reported in zebrafish in presumptive CNS and retinal neurogenesis and in proliferating NEP cell of developing neural tube, respectively [22, 120–122]. Several sox genes (*sox2*,* sox4a*,* sox9a*/*sox9b*,* sox10*,* sox11*,* sox14*,* sox21b*, and* sox32*) are injury induced since they are not expressed in the uninjured cord and are upregulated in 3 DPI, 10 DPI, and 15 DPI cord [23]. Temporal expression pattern and the highest fold change values in different time points for different sox genes suggest multiple roles during regeneration, which need to be validated separately. Only expression of* sox2* was validated and the data indicate presence of neural stem cell-like populations in adult regenerating cord, as these sox2 positive cells are proliferating [23, 91] similar to what has been found by others [123]. However, sequential actions of many sox genes are required for early pluripotential stem cells, for the generation of differential progeny and neurogenesis [124–126].

Proneural bHLH transcription factors are known to promote neurogenesis. Our data suggests that several proneural genes such as* ngn1*,* neurod2*,* neurod4*, and* olig2* are induced after SCI and may be involved in promoting differentiation of progenitors selectively to different neural fate. While* mash1*/*ascl1* is expressed after CNS injury in zebrafish [127, 128],* mash1* is required for gap-43 expression after optic nerve injury and ascl1a is required for retinal ganglion cell regeneration [128]. Role of* ascl1* in specification of GABAergic phenotypes during retinal neurogenesis in* Xenopus* is also reported. In developing spinal cord,* mash1* and* ngn2* are involved in specification of neuronal subtype identity [129, 130].* Ngn1* activity is required for sensory neuron development in zebrafish [131]. In regenerating cord, context dependent generation of specific neural cells types and role of different proneural genes can be explored further.

## 10. Signaling Pathways Involved in Repatterning

The mechanisms underlying the progenitor cell maintenance and neurogenesis are controlled by several signaling pathways such as Sonic Hedgehog (Shh), bone morphogenetic proteins (Bmp), Wnt signaling, and Fgfs. Our genome-wide expression profiling data indicated that several signaling pathways are involved in regeneration of adult spinal cord. These are Wnt, Bmp/Tgf beta, Hedgehog, Notch, and Fgf pathway [23]. Some of these signaling molecules are necessary for both regenerating and developing CNS. We observe upregulation of* shh* and* ptc1* in 3 DPI and 10 DPI cord. Others reported that ventral midline radial glia upregulate* shh* and* ptc1* repress adjacent P_MN_-like progenitors after injury. Involvement of* shh* is further confirmed by cyclopamine treated cord, where Hedgehog signaling was reduced and affected ventricular proliferation and impaired regeneration of motor neuron and serotonergic neurons [92, 132]. Although in the zebrafish embryonic cord Hedgehog signaling is not necessary for medial plate specification, several experiments reveal that the number of primary motor neurons is proportional to the level of Hedgehog signaling, since loss-of-function mutation for two out of three Hedgehogs has fewer primary motor neurons [19].

In developing zebrafish spinal cord, Bmp signaling establishes D-V pattern involving several mutants of Bmp pathways like Swirl/Bmp-2b, Snailhouse/Bmp-7, and somitabun/smad5 and it has been demonstrated that Bmp signaling is essential for establishing neural crest and RB neurons [131]. Severe depletion of BMP signaling by overexpression of nodal gene causes loss of RB as well as interneurons of spinal cord and expansion of ventral spinal cord fates. Bmp depletion in Swirl/Bmp-2 mutant leads to loss of RBs and expansion of interneurons. Thus, Bmp signaling suppresses formation of ventral cell types. During regeneration, members of Bmp/Tgf-*β* signaling pathways are differentially up- or downregulated and may be associated with different events. For example, growth and differentiation factor 11 (gdf11) is known to control neurogenesis in olfactory neuroepithelia [133, 134] and helps in maintaining progenitor population. Tgf-*β*1 expression is upregulated in early phases of regeneration in spinal cord suggesting its association with inflammatory response and proliferation [23].

Another important signaling pathway is Fgf signaling; in mammalian CNS, Fgf2 promotes neurogenesis after injury [87]. In zebrafish spinal cord, Fgf signaling promotes proliferation of radial glia and improves functional recovery [65].

Retinoic acid (RA) signaling is required for the generation of correct numbers of many different spinal neurons in developing zebrafish spinal cord, which basically affects cell proliferation. In the absence of Hedgehog and RA signaling, V_0_, V_1_, and V_2_ cells are formed, but Hedgehog signaling is required for the formation of V_3_ and P_MN_ domain cells [116]. Inhibition of RA signals perturbs tail regeneration in salamander [135]. After spinal cord injury, RA signaling cascade is activated by trauma. In embryonic tissues, RA can increase axonal outgrowth from spinal cord, DRG, and cerebellum [136–138].

Organization and function of spinal cord depend on developmental programs that determine proliferation and patterning in developing spinal cord. RP and FP are both considered to be the organizing centers secreting various morphogens like Wnt, Bmp, and Hedgehog proteins. In zebrafish canonical Wnt signaling is required for patterning and proliferation in the dorsal spinal cord. The* tcf7* is required for dorsal progenitor patterning, whereas* tcf3* (*tcf7l1*) regulates proliferation but not patterning [139]. The functions of canonical Wnt signaling in spinal cord cell proliferation and dorsal patterning are conserved in different vertebrate species, although Wnt targets may vary. Unlike amniotes, the dorsal limit of* dbx* expression in spinal cord is controlled by* wnt* in zebrafish and 24 hr postfertilized zebrafish spinal progenitors do not express bHLH family gene in the dorsal domain, indicating that individual dorsal patterning markers may be regulated by diverse mechanism downstream of Wnt signal. During development, rate of neurogenesis is controlled by extrinsic and intrinsic factors. Similarly, D-V subregions of progenitors are established by various signals such as Shh, Bmp, and Wnts as was discussed in the previous section.* Tcf7l1* plays a pivotal role in spinal cord progenitor maintenance and controls generation of neurons and glia from P_MN_ progenitor pool. Expression of* tcf7l1* is also required to inhibit the premature neurogenesis in spinal progenitor by repressing* sox4a*, a known mediator of spinal neurogenesis [140]. Expression of* tcf7l1* can be seen in larval zebrafish beyond primary neurogenesis [141].

Wnt contributes to adult neurogenesis, protects excitatory synaptic terminals from amyloid-*β* oligomer toxicity, and hence could be targeted for generation of potential therapy in neurodegenerative disorders like Alzheimer's and Parkinson's disease [142]. In regenerating zebrafish retina *β*-catenin/Wnt signaling controls the fate of the progenitors [143], where Wnt signaling is required for retinal neurogenesis. Following injury in retina, there are dedifferentiation and proliferation of Mullerian glia [144, 145]. These cells divide and generate neuronal progenitor and Wnt signal is required for glial dependent regeneration [146]. In regenerating zebrafish cord, many members of Wnt pathways are differentially regulated such as* wnt8a*,* wnt9a*,* wnt11*, and *β-catenin* [23]. Some of these genes are associated with proliferation like* wnt8a*, which is expressed in the ependymal cell following an injury. Both positive and negative regulators of Wnt signaling pathways like* dixdc*,* gsk3b*,* tcf7l1*,* tcf7l2*, and several* sfrp* are all differentially upregulated during regeneration of zebrafish spinal cord [23]. Role of Wnt signaling in neurogenesis during regeneration of spinal cord needs to be reexamined by using lineage tracing and functional assays.

## 11. Reprogramming and Epigenetic Program Controlling Regeneration

Zebrafish generate new neurons in the various parts of CNS like brain, spinal cord, and retina throughout their adulthood. Radial glia are progenitor cells in the developing mammalian CNS which exhibit neurogenic properties in adult zebrafish cord, suggestive of their regenerative capability. These radial glia are thought to be the adult equivalent of neuroepithelial cells. Upon injury to the zebrafish spinal cord and telencephalon, the radial glial cells display certain cellular responses which include (a) transient dedifferentiation, since these cells exhibit loss of glial markers; (b) asymmetric, self-renewing division; and (c) redifferentiation [13, 88]. Radial glia also share similar cellular and molecular properties of Muller glia [144, 145]. Based on several studies on different regeneration models, it has been hypothesized that tissue regeneration involves cellular reprogramming, like dedifferentiation and transdifferentiation. There are several other examples of regenerating organs, such as the zebrafish heart and the fin, where dedifferentiation occurs [147]. The dedifferentiation process has been reported to be related to the cell cycle reentry in most of the regenerating tissues.

There is an ongoing debate on whether the reprogramming of Muller glia/radial glia during regeneration is the right attribute or not [148, 149]. While some recognized that retinal regeneration is due to reprogramming, others concluded that Muller glia and radial glia both are actually multipotent stem cells that may not require “classical reprogramming.” In many of the regenerating organs mentioned above, there is replacement of lost or damaged cells that can be achieved by dedifferentiation, transdifferentiation, or reprogramming. Expression of only the four core transcription factors like sox2, oct4, klf4, and c-myc not only can restore pluripotency in a fully differentiated state, but also can make the cell adept to proliferate. Furthermore, introduction of three proneural transcription factors (brn2, ascl1, and myt1l) was shown to reprogram cells directly from fibroblast to specified neural phenotypes (e.g., differentiated and spinal motor neuron) bypassing the pluripotent intermediary [150, 151]. These observations indicate that the plasticity of the differentiated state may not be restricted to lower organisms and dedifferentiation program is rather more broadly demonstrated in all animals contrary to our previous belief.

The term reprogramming is commonly used to describe a process in somatic cells whereby they are required to undergo cell type reversal from a differentiated state to the pluripotent state with the consequent loss of differentiated identity. Reprogramming involves elimination and remodeling of epigenetic marks, such as DNA methylation and histone and chromatin structure modification. Evidence supporting epigenetic mechanism of neuroplasticity is still meager and epigenetic regulation in the process of neural regeneration is a promising concept. A recent report discussed selected epigenetic mechanisms controlling neuroplasticity after stroke [152]. Histone modifications are made by histone acyl transferases (HATs) and histone deacetylases (HDACs). HDAC inhibitors are known to promote neurogenesis and neuronal differentiation [153–155]. We observe that HDAC/HAT expression is differentially regulated in regenerating zebrafish cord [23], although epigenetic control of neural plasticity in regenerating zebrafish cord remains to be elucidated.

## 12. Strategies and Challenges for Neural Regeneration

SCI, stroke, and many other CNS disorders are characterized by a massive loss of neurons. Many of these conditions are debilitating and physically challenging because of loss of important functions. Damage to central nervous system can occur either from traumatic injury or through a neurodegenerative mechanism, but, irrespective of the cause, the ultimate outcome of the damage affects neurons and axons, resulting in an inability to conduct electrical impulse to different regions of the body. In order to revive such crucial functions of nervous system, one requirement for successful repair would be the regrowth of the damaged axons, while the neuronal cell bodies remain protected from damage. The other requirement is protection of the neural cells, generation of new neurons, and the replacement of cells lost due to an injury. Addressing these repair mechanisms would allow us to evolve potential strategies that would be essential to overcome the specific damage. Some of these strategies are accomplished by certain regions of nervous system like the peripheral nervous system (PNS), as well as within the CNS of lower vertebrates with greater regenerative capabilities than those of the mammals. In the future, a new therapeutic strategy needs to be developed and refined.

With recent advances in the technological front, we experience the merging of different areas such as developmental neurobiology, neurogenetics, genomics, live imaging, and physiology that allowed us to exploit zebrafish as a model organism for studying the CNS regeneration. One of the key factors that can influence the failure and/or success of regeneration includes neurogenesis in regenerating CNS. There are substantial lines of evidence to believe that, in regeneration of many adult tissues and organs, the existing molecular cues in development are redeployed. Thus, it is imperative to study the underlying molecular basis of neurogenesis during CNS development in order to uncover the molecular signals that are adapted during adult neurogenesis. In the present deliberation, we have tried to present an overview of current knowledge on adult neurogenesis and embryonic neurogenesis in zebrafish. These lines of evidence on neurogenesis would allow us to develop future therapeutic strategy to induce neurogenesis in adult higher vertebrates. The rapidly increasing knowledge on cell fate specification during embryogenesis and in adult lineages would aid in the development of experimental strategies to modulate endogenous stem/progenitor cells for spinal cord repair.

A promising strategy towards the restoration of function in a damaged CNS would be based on the induction of intrinsic regeneration potential of the CNS through the activation of endogenous neural progenitor or stem cells. Thus, it is important to identify the progenitors in the regenerating cord, so that their contribution to neurogenesis could be elucidated and appropriate therapy could be targeted to achieve functional recovery.

## Figures and Tables

**Table 1:** Neuroanatomy of human and zebrafish spinal motor system.

Similarities with human	Key differences and unknown features
*Spinal cord *	
Spinal motor neurons	
(a) Zebrafish SMN similar to human *α*-MN.	Absence of *γ*-MN in zebrafish.
(b) Presence of different subtypes of MN, some located at specific regions of the spinal cord, with some at specific region of the cord, innervating axial and fin muscle.	P_MNs_ have not been reported in human. Appendicular MNs in fish are not separated into a LMC.
Skeletal muscle fibres	
(a) Similar morphological, molecular, and histological features like dystrophin associated glycoprotein complex, excitation-contraction coupling, and contractile machinery.	Proprioceptors like muscle spindle are absent in fish.

*Brainstem*	
Ventromedial brainstem descending pathway	
(a) Fibres from RF, VN, and nMLF descend from hind brain along with MFL through spinal cord as VMF, projecting onto interneurons and some spinal MN.	Human brainstem contains UMN. Fibres from RF, VN, and SuC descend to MMC of spinal cord projecting onto interneuron and spinal motor neuron. A direct tectospinal tract has not been identified.
Dorsolateral brainstem descending pathway	A few rubrospinal fibres present in zebrafish; a true rubrospinal tract is absent.

*Motor cortex*	
Corticospinal tract (CST)	No CST in teleost fish.

P_MN_: primary motor neuron (CaP, MiP, RoPs, and VaP); SMN: secondary motor neuron (vS: ventrally projecting SMNS); LMC: dorsal lateral motor column; RF: reticular formation; VN: vestibular nuclei; SuC: superior colliculus; nMLF: nucleus of the medial longitudinal fasciculus; VMF: ventromedial fascicle; MN: motor neuron; UMN: upper motor neuron; MMC: ventral medial motor column; CST: corticospinal tract. Adapted and modified from [41].

**Table 2:** Transcription factors expressed in zebrafish ventral spinal cord.

Progenitor domains in spinal cord	Expressed transcription factors
DP_5_	*irx3a*, *gsx1*, *pax7a*
DP_6_	*irx3a*, *dbx2*, *pax7a*
P_0_	*irx3a*, *dbx2*, *dbx1a*
P_1_	*irx3a*, *dbx2*, *nkx6.2*
P_2_	*irx3a*; *nkx6.1*, *nkx6.2*
P_MN_	*olig2*, *nkx6.1*, *nkx6.2, islet2a*
P_3_	*nkx2.2b*, *nkx6.1*, *nkx6.2*
V_0_	*pax2a*, *evx1*
V_1_	*pax2a*, *eng1b*
V_2_	*gata3 *(V_2b_), *vsx1/2 *(V_2a_)
V_3_	*gata3*, *tal2*

DP: dorsal progenitor; P: progenitor; V: ventral progenitor.
